# Breakdown of oscillatory effective networks in disorders of consciousness

**DOI:** 10.1111/cns.14469

**Published:** 2023-09-17

**Authors:** Yang Bai, Anjuan Gong, Qijun Wang, Yongkun Guo, Yin Zhang, Zhen Feng

**Affiliations:** ^1^ Department of Rehabilitation Medicine The First Affiliated Hospital of Nanchang University Nanchang China; ^2^ Rehabilitation Medicine Clinical Research Center of Jiangxi Province Nanchang China; ^3^ Center for Cognition and Brain Disorders The Affiliated Hospital of Hangzhou Normal University Hangzhou China; ^4^ The Fifth Affiliated Hospital of Zhengzhou University Zhengzhou China

**Keywords:** disorders of consciousness, effective network, oscillatory reactivity, TMS‐EEG

## Abstract

**Introduction:**

Combining transcranial magnetic stimulation with electroencephalography (TMS‐EEG), oscillatory reactivity can be measured, allowing us to investigate the interaction between local and distant cortical oscillations. However, the extent to which human consciousness is related to these oscillatory effective networks has yet to be explored.

**Aims:**

We tend to investigate the link between oscillatory effective networks and brain consciousness, by monitoring the global transmission of TMS‐induced oscillations in disorders of consciousness (DOC).

**Results:**

A cohort of DOC patients was included in this study, which included 28 patients with a minimally conscious state (MCS) and 20 patients with vegetative state/unresponsive wakefulness syndrome (VS/UWS). Additionally, 25 healthy controls were enrolled. The oscillatory reactivity to single‐pulse TMS of the frontal, sensorimotor and parietal cortex was measured using event‐related spectral perturbation of TMS‐EEG. The temporal–spatial properties of the oscillatory reactivity were illustrated through life time, decay gradients and accumulative power. In DOC patients, an oscillatory reactivity was observed to be temporally and spatially suppressed. TMS‐EEG of DOC patients showed that the oscillations did not travel as far in healthy controls, in terms of both temporal and spatial dimensions. Moreover, cortical theta reactivity was found to be a reliable indicator in distinguishing DOC versus healthy controls when TMS of the parietal region and in distinguishing MCS versus VS/UWS when TMS of the frontal region. Additionally, a positive correlation was observed between the Coma Recovery Scale‐Revised scores of the DOC patients and the cortical theta reactivity.

**Conclusions:**

The findings revealed a breakdown of oscillatory effective networks in DOC patients, which has implications for the use of TMS‐EEG in DOC evaluation and offers a neural oscillation viewpoint on the neurological basis of human consciousness.

## Graphical abstract

We revealed a temporally and spatially breakdown of oscillatory effective network in disorders of consciousness, by measuring oscillatory reactivity to transcranial magnetic stimulation. This provides a new insight into the part of brain oscillation networks in disorders of consciousness and furthers our comprehension of neurophysiological foundations of human consciousness.

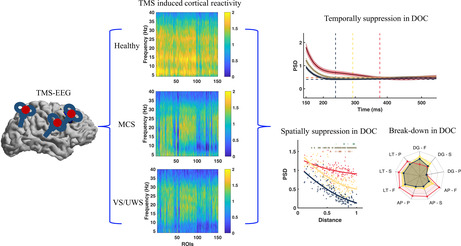

## 1 INTRODUCTION

An injury or dysfunction of the neural systems regulating arousal and awareness might cause an altered consciousness state.^1^, ^2^ Patients with the absence of arousal (i.e., eye opening even when stimulated) and awareness (unaware of themselves and the environment) were diagnosed with a disorder of consciousness (DOC).^3^ DOC, consisting of a vegetative state/unresponsive wakefulness syndrome (VS/UWS)^4^ and a minimally conscious state (MCS),^5^ is an important but still underexplored entity in neurology. Clinicians and neuroscientists are increasingly turning to neuroimaging and electrophysiological measurements to gain a better understanding of human consciousness, obtaining brain information of DOC in the process.

Cortical oscillations have been extensively studied, and certain frequency bands have been linked to particular brain functions, as well as to certain cortical and sub‐cortical regions.^6^, ^7^ Changes in the properties of cortical and sub‐cortical (especially thalamic) neurons, as well as changes in their patterns of connectivity, underlie most neurological and psychiatric conditions and may lead to distinctive and detectable changes in their oscillatory properties.^7^, ^8^ In EEG, highly abnormal brain oscillations are the typical biomarkers of DOC.^9^ Generally, a low level of consciousness is associated with an increased slow and suppressed fast spontaneous oscillation.^10^ Due to their close relationship with the preserved consciousness function^10^, ^11^, ^12^ and outcomes of DOC,^9^, ^13^ brain oscillations have been gaining more attention from clinical and research circles related to DOC.

Most research on brain oscillations focus on spontaneous EEG activity; nevertheless, a more accurate estimate of the oscillation tuning of cortical areas can be attained by measuring steady‐state‐evoked responses. It is theorized that TMS interacts with both nearby and distant cortical oscillations. As such, the combination of TMS and EEG provides the opportunity to measure activity in frequency bands directly induced by the TMS pulse, which is depicted as a transient phase alignment of the oscillatory activity.^14^, ^15^ The added value of TMS‐EEG in the study of cortical oscillations is the possibility to study the functional specificity of brain rhythms,^16^ as well as to probe thalamocortical circuits via direct measurement of TMS‐induced oscillatory activity that results from phase‐reset by TMS.^17^, ^18^, ^19^ The oscillatory cortical reactivity to TMS has proven to be a valuable technique to characterize the oscillatory activity of a brain area,^17^ discriminate between normal and clinical cortical oscillatory patterns,^20^, ^21^ investigate changes caused by experimental manipulative approaches^22^, ^23^ and evaluate the causal role of specific oscillatory network activity during task execution.^24^

Since TMS‐EEG does not rely on the integrity of motor pathways (in contrast to TMS‐evoked electromyography from muscle) and does not require any active participation (in contrast to event‐related‐potentials) from the patient, it is now considered one of the most promising techniques in the diagnosis of DOC patients.^9^ The variations of TMS evoked potentials in different consciousness levels have been well‐defined recently.^25^ However, to date, no research has been conducted to investigate the oscillatory reactivity of the brain in the state of DOC when directly stimulated at different cortical sites. By tracking the global spread of TMS‐induced oscillatory waves, we can gain insight into the oscillatory effective networks, which could help to better understand the mechanisms of human consciousness by detecting causal interactions, information transmission and the state of the brain's system. To this end, we conducted a TMS‐EEG study to explore the oscillatory reactivity in DOC patients, hypothesizing a breakdown of the oscillatory reactivity network in the unconscious brain.

## 2 METHODS

### 2.1 Participants

DOC patients were enrolled into the study based on the inclusion criteria: (i) persistence of a DOC state for at least 1 month after the acute brain insult; (ii) stable vital signs and free of acute medical complications (e.g., acute pneumonia). Exclusion criteria included any contraindications to MRI or TMS (e.g., pacemakers, intracranial stents, epilepsy, incomplete skull) and no detectable (peak‐to‐peak amplitude <50 μV with maximum output of TMS) motor evoked potentials (MEP) in hand muscles with TMS of the primary motor cortex (M1) of either hemisphere, even at maximum stimulator output. Finally, a cohort of 48 DOC patients (21 females; mean age ± SD: 49.58 ± 15.61 years) (Table S1) were included in the study. Twenty‐five age‐matched healthy subjects (10 females and 15 males; mean age ± SD: 42.12 ± 10.18 years) were included to form the control group for this study. All participants were screened for possible contraindications to TMS and met the criteria specified in the TMS safety screening questionnaire.^26^ Informed consent to participate in the study was obtained from the legal custodians of the DOC patients and healthy participants. This study was approved by the Ethics Committee of the Affiliated Hospital of Hangzhou Normal University (protocol number 20200320‐BDC07).

### 2.2 Consciousness diagnosis

The consciousness diagnosis of the DOC patients was conducted by well‐trained neurologists using the Coma Recovery Scale‐Revised (CRS‐R),^3^ which includes six items that test auditory, visual, motor, oromotor, communication, and arousal function. The CRS‐R scale is both quantitative (scores ranging from 0 to 23, with higher scores indicating a higher level of neurobehavioral function) and qualitative, as it provides sub‐scores that define different states of consciousness (MCS or UWS/VS). The CRS‐R assessment was performed at least four times within 1 week. The clinical diagnosis was established according to the best CRS‐R scores to counteract the impacts of fluctuating consciousness levels. Accordingly, 28/48 (age mean ± 1SD: 49.71 ± 15.75 years, females = 12) of DOC patients were diagnosed as MCS and 20/48 (age mean ± 1SD: 49.40 ± 15.83 years, females = 9) patients were diagnosed as UWS/VS;

### 2.3 TMS‐EEG data recording

Individual anatomical MRI (three‐dimensional gradient echo T1) was acquired from the patients in a clinical routine setting. TMS‐EEG data were acquired by a TMS‐compatible EEG system (BrainAmp 64 MR Plus, BrainProducts GmbH). The EEG cap was equipped with TMS‐compatible C‐ring slit Ag/AgCl pin electrodes arranged in the International 10–20 montage. The EEG amplifier was set with a hardware filter at DC to 10 kHz and a sampling rate of 5 kHz. The skin/electrode impedances of all electrodes were maintained below 5 kΩ throughout the data recordings. TMS pulses were delivered through a 70 mm figure‐of‐eight coil connecting a Magstim *R* ^2^ stimulator (Magstim Company Limited) with a monophasic current waveform. Participants were seated on a comfortable reclining rehabilitation chair and maintained an arousal state (eyes open) during the recordings. A CRS‐R arousal facilitation protocol was performed to keep the patients aroused during TMS‐EEG recording.

A BrainSight neuronavigation system (Rogue Research Inc.) was used to enable consistent positioning of TMS coil. To define the standardized localization of TMS targets, the participants' head was co‐registered to individual MRI data and was mapped to the Montreal Neurological Institute (MNI) coordinate system. Three cortical markers were set according to the MNI coordinates (frontal: *x* = 43, *y* = 21, *z* = 38; sensorimotor: *x* = 50, *y* = −7, *z* = 56; parietal: *x* = 30, *y* = −67, *z* = 60) on the individually MNI‐fitted images. The corresponding coil positions were set on the surface, tangential to the scalp and determined, orienting the coil such that the induced electric field was perpendicular to the main axis of the target gyrus.

Prior to the TMS‐EEG recordings, the individual resting motor threshold (RMT) was determined by motor‐evoked potential (MEP) recording from the abductor pollicis brevis muscle using surface EMG Ag‐AgCl cup electrodes in a belly‐tendon montage. The EMG raw signal was amplified, bandpass filtered (20 Hz–2 kHz) with a 2‐channel EMG device built in the Brain Sight system. Individual RMT was defined as the minimum intensity that was sufficient to elicit an MEP of >50 μV peak‐to‐peak amplitude in at least five out of ten subsequent trials.^27^

200 single TMS pulses at an intensity of 90% RMT were delivered with jittered inter‐trial intervals of on average 2.5 s at each target. The order of the targets was pseudo‐randomized and balanced across the participants. The participants were asked to wear earphones with white noise during the TMS‐EEG recordings to avoid EEG contamination by auditory evoked potentials caused by the TMS coil click.^28^

### 2.4 Data processing and analysis

The MRI pre‐processing and mesh extraction were performed based on the FieldTrip toolbox running in the MATLAB (Version 2017b, MathWorks Inc.) environment and FreeSurfer, following the pipeline of our previous study.^29^ Preprocessing of TMS‐EEG was performed using customized analysis scripts on MATLAB and EEGLAB 14.1.2b. The continuous TMS‐EEG data were segmented into epochs with respect to the TMS trigger markers. The epochs were defined from −1000 to 1000 ms and baseline corrected with −500 to −20 ms. Data from −2 to 10 ms around the TMS pulses were excluded and cubic interpolated to eliminate the high‐amplitude TMS artifact. EEG data were then visually inspected to identify and exclude the epochs containing major artifacts and the channels that showed prominent noise in most of the epochs. Afterward, data were down‐sampled from 5 to 1 kHz and submitted to a two‐step ICA procedure. In the first step, ICA components representing high‐amplitude TMS‐related artifacts were inspected and removed based on the topography, single‐trial time‐course and average time‐course. Then, data were filtered with a 1–100 Hz zero‐phase Butterworth band‐pass filter and a 48–52 Hz notch filter. As a second step, ICA was used to remove artifacts containing eye blinks, eye movement and persistent scalp muscle activity. Finally, the channels that were discarded during the visual inspection were spline‐interpolated using the signal of the neighbor channels and data were then average referenced.

The individual lead field matrix was calculated based on the aligned electrodes, individual mesh, and head model. The source reconstruction of cortical responses was performed using a linearly constrained minimum variance beamforming method.^30^

### 2.5 Event‐related spectral perturbation and oscillatory reactivity properties

We performed event‐related spectral perturbation (ERSP) analysis of the TMS evoked potentials (TEPs) to explore the oscillatory reactivity induced by TMS. Time‐frequency representations (TFRs) of TMS‐related oscillatory power were calculated, separately for each region of interest (ROI) at the single trial level, by means of a Hanning taper windowed FFT with frequency‐dependent window length (width: 3.5 cycles per time window, time steps: 10 ms, frequency steps: 0.25 Hz from 4 to 45 Hz). Data from −1000 to 1000 ms around the TMS pulse were selected to ensure a sufficient time and frequency resolution of the ERSPs. A cluster‐permutation process (shuffle, 200 times, significance level < 0.05) was performed to exclude non‐significant induced oscillatory responses at each frequency with respect to baseline. Then, the ERSPs were extracted by cropping the TFRs during the time of interest (−600 to 600 ms) for further statistical analysis. The ERSPs of the source space dipoles were then projected into the human Brainnetome Atlas.^31^ The atlas contains 246 ROIs across both hemispheres, including cortical and sub‐cortical regions. We extracted the cortical ROIs (157/246). The oscillatory reactivity of ROIs was extracted by averaging ERSPs of dipoles included in the ROIs.

In order to investigate the temporal–spatial properties of the oscillatory reactivity to TMS, we measured life time (LT), decay gradients (DG) and accumulative power (AP) of the oscillatory reactivity in the defined frequency of interests. Specifically, the life time was defined as the lasting time of the oscillatory reactivity following TMS pulses, which was determined by setting significant thresholds of the oscillatory power from the ERSP baseline. The decay gradients were measured by absolute average step gradients of the generalized linear regression of oscillatory power with normalized distance of ROIs to TMS targets. Accumulative power was measured by summarizing oscillatory reactivity power within life time of each ROI.

### 2.6 Statistical analysis

To uncover the temporal–spatial features of the oscillatory reactivity, we averaged the oscillatory power spectral density (PSD) values of the ERSPs in relevant frequency bands and studied the life time, decay gradients and the accumulative oscillatory reactivity power in ROIs. A bootstrap sampling statistic was used to determine significant thresholds of oscillatory activity.^32^ Specifically, a surrogate average baseline ERSP was obtained by shuffling the PSD values during pre‐TMS baseline (−300 to −50 ms) of single‐trial TEPs for each participant. The maximum absolute value distribution from the 1000 times shuffled surrogates was obtained to determine the thresholds of significant activity in post‐TMS periods (20–600 ms) with a significance level of *p* < 0.01. The life time of TMS‐induced oscillatory reactivity was determined by the amount of time above the significant thresholds. The distance between the ROIs and TMS targets was measured by Euclidean distance in MNI coordinates and normalized by the maximum and minimum values of all ROIs. The relationship between oscillatory reactivity power and spatial distance was examined using logistic regression.

We conducted two‐way repeated measures of ANOVA (rmANOVA) using the software SPSS (version 25) to test the significance of life time, decay gradients and accumulative power of oscillatory reactivity, after verifying the normal distribution of data by Shapiro–Wilk tests. The repeated effect of TMS targets (3 levels: frontal, sensorimotor, and parietal) and the effect of the group (3 levels: healthy, MCS, and UWS/VS) were investigated. Post‐hoc independent‐sample two‐tailed *t*‐tests were performed in case of a significant main effect of the group. *p* values were FDR‐corrected for multiple comparisons. The statistical tests of PSD values in group contrasts (healthy vs. MCS, healthy vs. UWS/VS, MCS vs. UWS/VS) along with frequency or distance were conducted by independent‐sample two‐tailed *t*‐tests, after verifying normal distribution of data. *p* values were FDR corrected based on the number of comparisons (289 times along frequency domain; 157 times along spatial distance).

Radar plots, receiver operating characteristic curve (ROC) and area under ROC (AUC) were used to show the classification capacity of oscillatory reactivity properties in group contrasts. We conducted a stepwise logistic regression and a Wald statistic to determine the strongest predictor of CRS‐R values among all the oscillatory reactivity properties. Pearson correlation analyses and generalized line regressions with scatter plots were used to examine the relationship between the best predictor and CRS‐R values.

## 3 RESULTS

**FIGURE 1:**
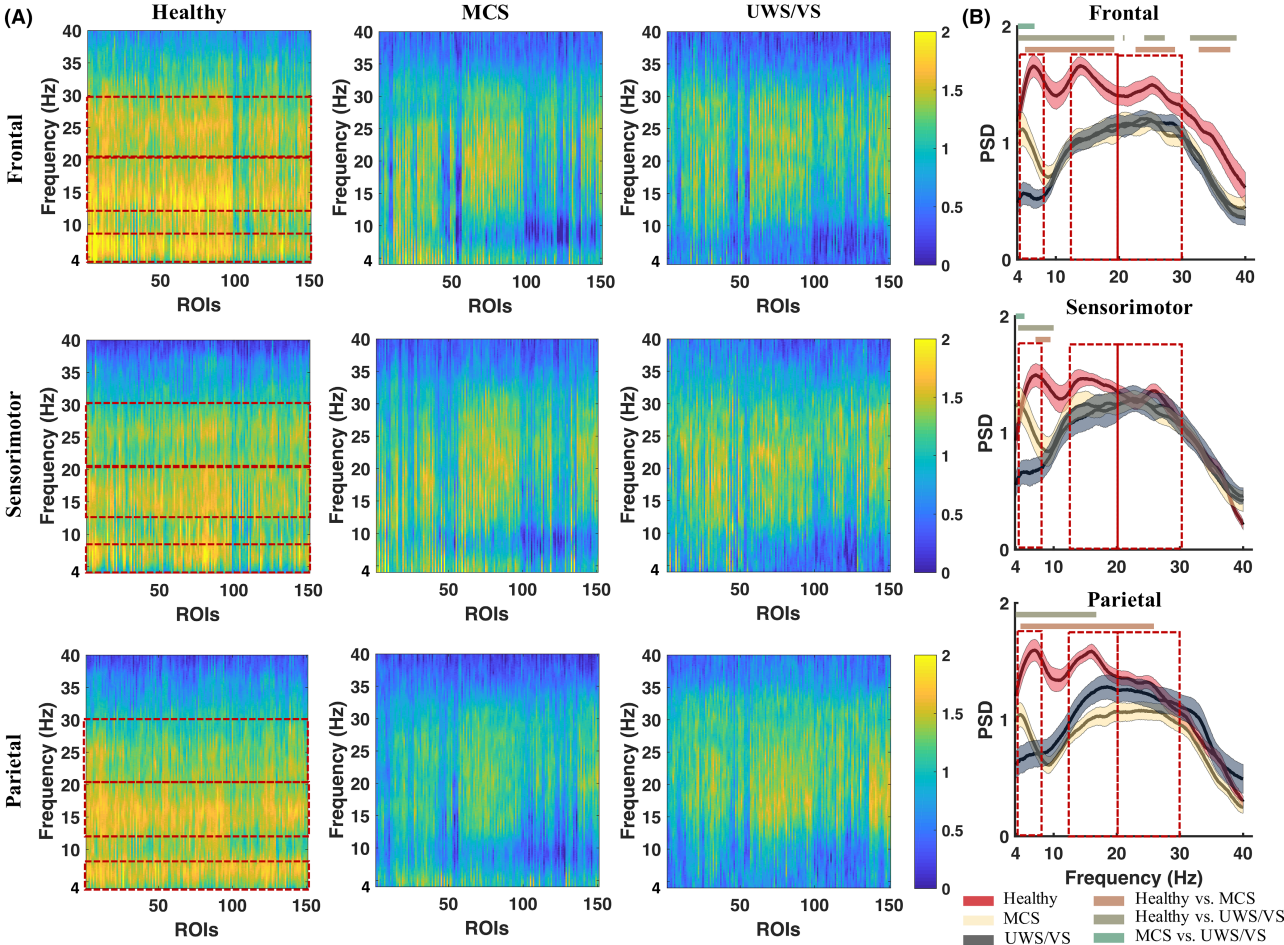
Cortical oscillatory reactivity with transcranial magnetic stimulation (TMS) at frontal, sensorimotor and parietal in healthy, minimally conscious state (MCS) and unresponsive wakefulness syndrome/vegetative state (UWS/VS). (A) Oscillatory reactivity in frequency with brain regions of interest (ROIs), measured by the average power spectral density (PSD) from 20 to 300 ms in healthy (left), MCS (middle) and UWS/VS (right), with frontal (upper), sensorimotor (middle), and parietal (lower) TMS. (B) Average PSD (mean ± 1SEM) of each frequency across all ROIs in healthy (red), MCS (yellow), and UWS/VS (gray). The heavy lines above curves indicate statistical significance (independent *t*‐tests with FDR correction, *p* < 0.05) in group contrasts. Red dotted boxes show frequency bands of interests, correspondingly defined as theta (4–8 Hz), low‐beta (13–20 Hz), and fast‐beta (21–30 Hz).

ERSPs were acquired from 157 cortical ROIs of 25 healthy induviduals, 28 MCS patients and 20 UWS/VS patients, with frontal, sensorimotor and parietal TMS. TMS caused distinct oscillatory reactivity, mainly in theta and beta bands, spreading across brain regions (with an average of 20–300 ms of ERSPs) in the healthy group (Figure 1A). The healthy group demonstrated significantly greater (two‐tailed independent *t*‐tests, with *p* < 0.05 after FDR correction) broad band (4–40 Hz) oscillatory reactivity (average of ROIs) with frontal TMS, theta–alpha band (healthy vs. MCS: 7–10 Hz; healthy vs. UWS/VS: 4–10 Hz) reactivity with sensorimotor TMS, and theta–alpha–beta band reactivity (healthy vs. MCS: 5–26 Hz; healthy vs. UWS/VS: 4–18 Hz) with parietal TMS (Figure 1B), compared to the DOC group. The MCS group showed exhibited a greater theta reactivity than UWS/VS when exposed to frontal and sensorimotor TMS. Three frequency bands were highlighted in the healthy group, which were characterized by their wide spatial distribution and spectral dominance (Figure 1A,B). Frequency bands of interest (theta: 4–8 Hz; low‐beta: 13–20 Hz; fast‐beta: 21–30 Hz) were determined accordingly. The temporal–spatial properties of the TMS‐induced oscillatory reactivity were then examined within the specified bands.

**FIGURE 2:**
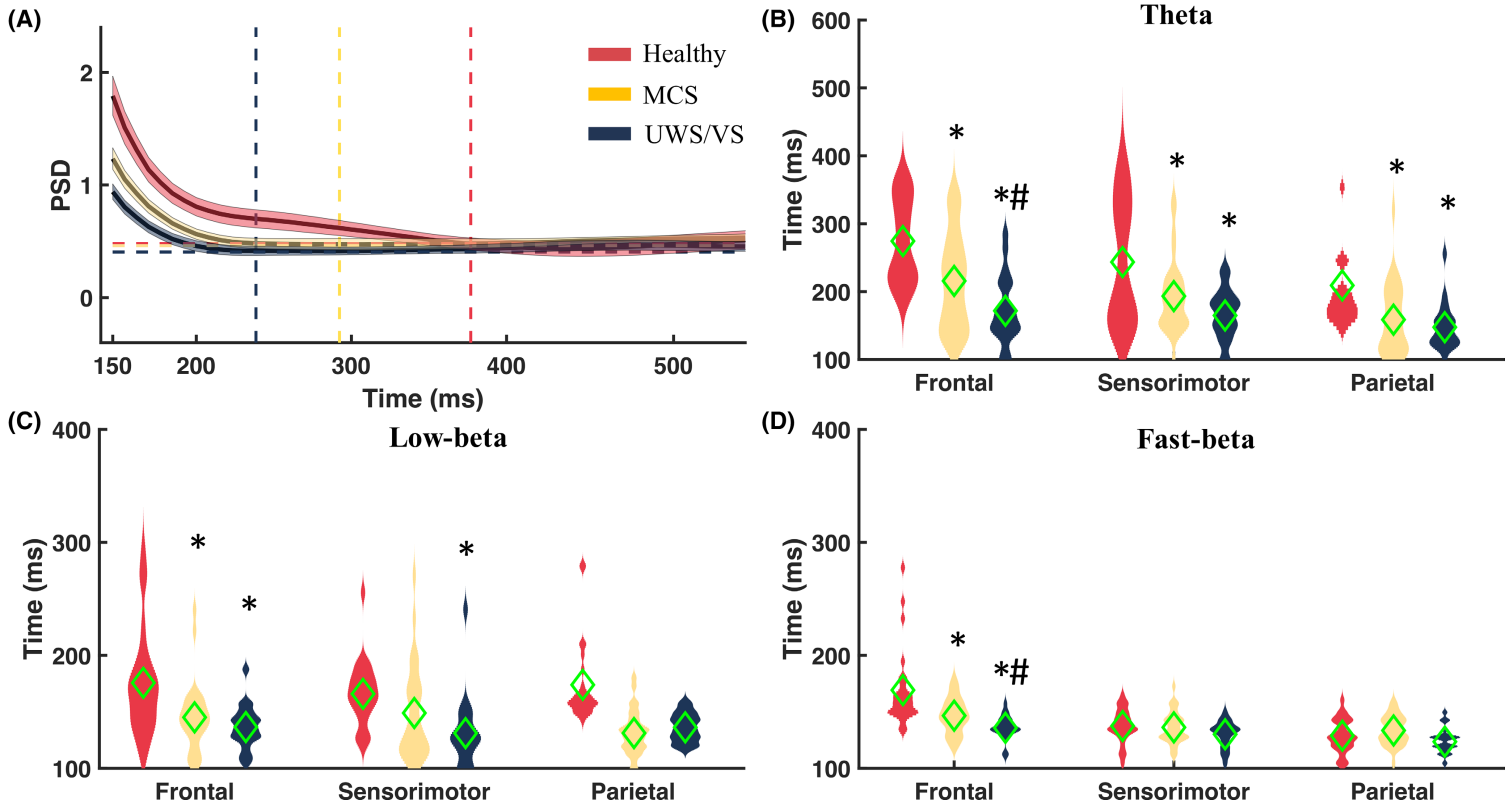
Life time of oscillatory reactivity to transcranial magnetic stimulation (TMS). (A) Oscillatory reactivity (average of 4–40 Hz across all brain regions) in healthy subjects (red, mean ± SEM), patients with minimally conscious state (MCS) (yellow) and unresponsive wakefulness syndrome/vegetative state (UWS/VS) (gray). Horizontal dotted lines indicate significant (bootstrap permutation statistic with *p* < 0.05) thresholds from baseline of healthy (red), MCS (yellow), and UWS/VS (gray). Vertical dotted lines with corresponding *x*‐axis values indicate mean life time of the oscillatory reactivity in each group. The distribution plots show life time of oscillatory reactivity in defined theta (B), low‐beta (C), and fast‐beta (D) bands (average across of brain regions), induced by frontal, sensorimotor and parietal TMS in healthy, MCS and UWS/VS. Green diamonds represent mean values of groups. * indicates significant difference (two‐way repeated measure ANOVA, post‐hoc *t*‐tests with FDR correction, *p* < 0.05) between healthy versus MCS or healthy versus UWS/VS. # indicates significant difference between MCS and UWS/VS patients.

The average oscillatory reactivity (4–40 Hz across all ROIs) to TMS in healthy individuals lasts longer (mean life time = 383 ms) than in DOC groups (mean life time: MCS = 290 ms; UWS/VS = 245 ms) (Figure 2A). Two‐way rmANOVA disclosed the group main effect (*F*_(2, 210)_ = 26.330, *p* < 0.001), with a significantly longer life time of theta reactivity in healthy than MCS and UWS/VS, and a longer life time in MCS than UWS/VS, without interaction with TMS targets (*F*_(4, 210)_ = 0.601, *p* > 0.05). Post hoc *t*‐tests revealed a between‐group difference with significantly (all *p* < 0.01) longer theta reactivity in healthy group than MCS (mean ± 1SD of healthy vs. MCS with TMS at frontal: 274.46 ± 62.02 vs. 215.75 ± 70.08; sensorimotor: 243.54 ± 91.63 vs. 193.41 ± 55.07; parietal: 209.16 ± 102.77 vs. 158.72 ± 51.96) and UWS/VS group (with TMS at frontal: 171.76 ± 51.11; sensorimotor: 164.91 ± 38.26; parietal: 147.62 ± 34.90) (Figure 2B). MCS showed significantly longer theta reactivity than UWS/VS (*p* < 0.01). Group main effects were both significance in low‐beta (*F*_(2, 210)_ = 27.387, *p* < 0.001) and fast‐beta (*F*_(2, 210)_ = 12.669, *p* < 0.001) frequency bands. Post hoc *t*‐tests revealed longer low‐beta reactivity in the healthy group than MCS (175.71 ± 46.53 vs. 145.15 ± 30.51, *p* = 0.006) with frontal TMS, and than UWS/VS (frontal: 136.58 ± 19.15, *p* = 0.001; sensorimotor: 131.17 ± 32.79, *p* < 0.001) with frontal and sensorimotor TMS (Figure 2C). Fast‐beta reactivity showed a longer life time in the healthy group than MCS (169.26 ± 34.90 vs. 146.64 ± 16.00, *p* = 0.003) and UWS/VS group (135.91 ± 10.92, *p* < 0.001) only with frontal TMS (Figure 2D). The MCS group had significantly longer fast‐beta reactivity than the UWS/VS group with frontal TMS (*p* = 0.01).

**FIGURE 3:**
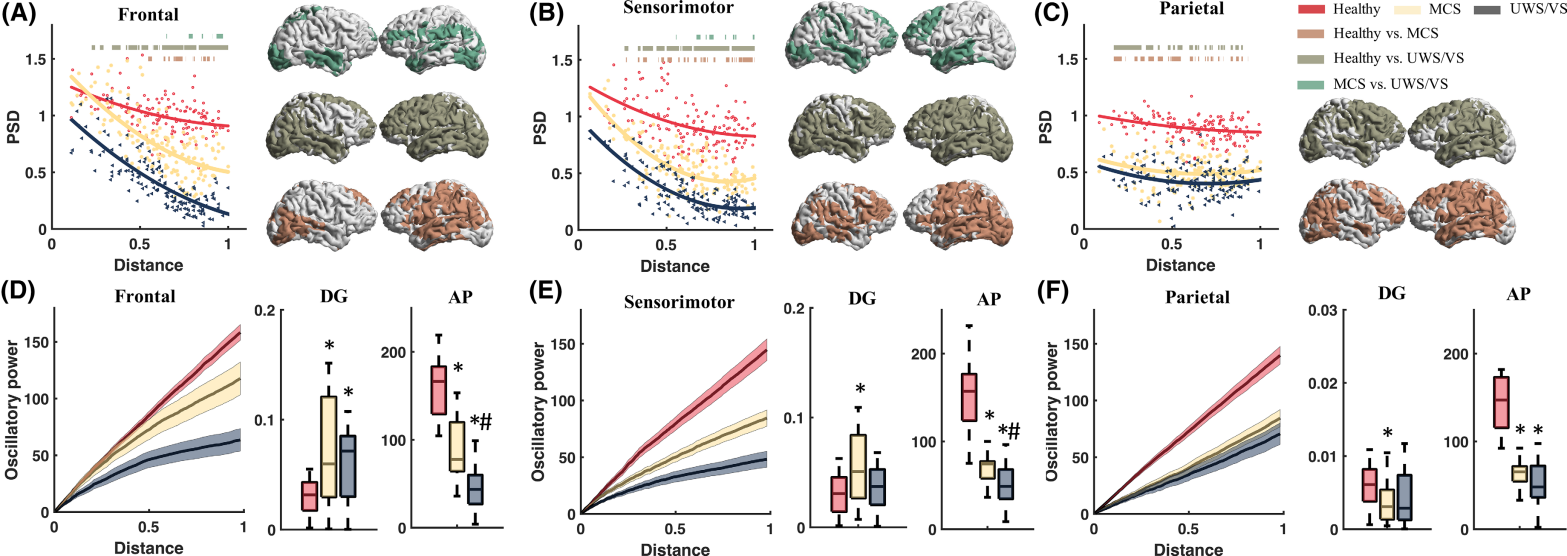
Cortical theta (4–8 Hz) reactivity to transcranial magnetic stimulation (TMS) in brain regions of healthy, minimally conscious state (MCS) and unresponsive wakefulness syndrome/vegetative state (UWS/VS). Scatter plots show theta power spectral density (PSD) of brain regions with normalized distance to stimulus targets with frontal (A), sensorimotor (B), and parietal (C) TMS of healthy (red), MCS (yellow), and UWS/VS (gray). Curves depict logistic regression between theta PSD and normalized distance. The heavy lines upper curves and spatial distribution maps at right side show statistical significance (independent two‐tails *t*‐tests with FDR correction, *p* < 0.05) in group contrasts. Accumulating theta reactivity power (mean ± SEM) along with normalized distance of brain regions to stimulus targets, with frontal (D), sensorimotor (E), and parietal (F) TMS. Boxplots show the power decay gradients (DG) and accumulative power (AP), which was significantly different between healthy (red), MCS (yellow) and UWS/VS (gray) groups. * indicates significant difference (independent two‐tails *t*‐tests with FDR correction, *p* < 0.05) between healthy versus MCS or healthy versus UWS/VS. # indicates significant difference between MCS and UWS/VS groups.

Theta reactivity decreased as the distance of ROIs to TMS targets increased (Figure 3A–C). In comparison to the healthy group, the MCS and UWS/VS group demonstrated significantly (two‐tailed independent *t*‐tests, with *p* < 0.05 after FDR correction) lower theta reactivity power in ROIs close to the frontal and sensorimotor TMS targets (Figure 3A,B), and in most of the ROIs with parietal TMS (Figure 3C). Additionally, the MCS group showed a significantly higher theta reactivity compared to UWS/VS in ROIs near the frontal and sensorimotor TMS targets, without a significant difference when it came to parietal TMS. Statistic tests revealed the significance of group main effects (*F*_(2, 210)_ = 13.995, *p* < 0.001) in decay gradients of theta reactivity, and significantly interacted with TMS targets (*F*_(4, 210)_ = 5.688, *p* < 0.001). Significant group main effects (*F*_(2, 210)_ = 264.263, *p* < 0.001) were found in accumulative theta power, with significantly larger theta accumulative power in the healthy group than MCS and UWS/VS, and larger in MCS than UWS/VS, without interaction (*F*_(4, 210)_ = 1.860, *p* > 0.05) with TMS targets. Post hoc *t*‐tests disclosed a between group difference with significantly higher decay gradients in DOC groups than healthy group with frontal (healthy vs. MCS: 0.030 ± 0.015 vs. 0.072 ± 0.050, *p* = 0.002; and vs. UWS/VS: 0.059 ± 0.034, *p* = 0.004) and sensorimotor TMS (healthy vs. MCS: 0.031 ± 0.018 vs. 0.056 ± 0.032, *p* = 0.001) (Figure 3D,E), and significantly lower decay gradients in MCS than the healthy group with TMS of parietal (healthy vs. MCS: 0.006 ± 0.003 vs. 0.004 ± 0.003, *p* = 0.007) (Figure 3F). Accumulative theta power of healthy group was significantly (all *p* < 0.001) higher than MCS and UWS/VS with frontal (healthy vs. MCS: 159.56 ± 32.68 vs. 88.07 ± 32.46; and vs. UWS/VS: 43.68 ± 26.11), sensorimotor (healthy vs. MCS: 151.89 ± 37.89 vs. 70.53 ± 13.78; and vs. UWS/VS: 48.17 ± 29.70) and parietal TMS (healthy vs. MCS: 145.81 ± 30.12 vs. 64.71 ± 12.87; and vs. UWS/VS: 50.71 ± 29.38). MCS showed significantly higher accumulative theta power than UWS/VS with frontal (*p* = 0.001) and sensorimotor TMS (*p* = 0.01) (Figure 3D,E).

**FIGURE 4:**
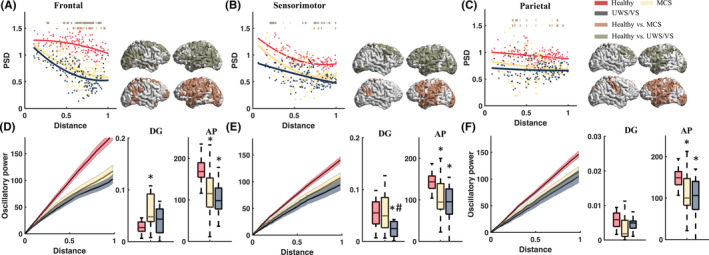
Cortical low‐beta (13–20 Hz) reactivity to transcranial magnetic stimulation (TMS) in brain regions of healthy, minimally conscious state (MCS) and unresponsive wakefulness syndrome/vegetative state (UWS/VS). Scatter plots show low‐beta power spectral density (PSD) of brain regions with normalized distance to stimulus targets with frontal (A), sensorimotor (B), and parietal (C) TMS in healthy (red), MCS (yellow), and UWS/VS (gray). Curves depict logistic regression between theta PSD and normalized distance. The heavy lines upper curves and spatial distribution maps at right side show statistical significance (independent two‐tails *t*‐tests with FDR correction, *p* < 0.05) in group contrasts. Accumulating low‐beta reactivity power (mean ± SEM) along with normalized distance of brain regions to stimulus targets, with frontal (D), sensorimotor (E), and parietal (F) TMS. Boxplots show the power decay gradients (DG) and accumulative power (AP), which was significantly different between healthy (red), MCS (yellow) and UWS/VS (gray) groups. * indicates significant difference (independent two‐tails *t*‐tests with FDR correction, *p* < 0.05) between healthy versus MCS or healthy versus UWS/VS. # indicates significant difference between MCS and UWS/VS patients.

The healthy group displayed significantly (two‐tailed independent *t*‐tests, with *p* < 0.05 after FDR correction) higher low‐beta reactivity power than MCS and UWS/VS group in certain distance ROIs (Figure 4A,B). Two‐way rmANOVA revealed the significance of group main effects (*F*_(2, 210)_ = 11.357, *p* < 0.001) in decay gradients of low‐beta reactivity, with significant interaction with TMS targets (*F*_(2, 210)_ = 8.935, *p* < 0.001). The healthy group showed significantly lower decay gradients than MCS (healthy vs. MCS: 0.028 ± 0.012 vs. 0.060 ± 0.033, *p* < 0.001) with frontal TMS (Figure 4D). UWS/VS group showed significantly lower decay gradients than the healthy group (healthy vs. UWS/VS: 0.054 ± 0.027 vs. 0.023 ± 0.015, *p* < 0.001) and MCS group (0.037 ± 0.023, *p* = 0.004) with sensorimotor TMS (Figure 4E). Significant group main effects (*F*_(4, 210)_ = 31.705, *p* < 0.001) were found in accumulative low‐beta power, with significantly larger low‐beta accumulative power in the healthy group than in DOC groups, without interaction (*F*_(4, 210)_ = 1.860, *p* > 0.05) with TMS targets. Post hoc *t*‐tests disclosed a between‐group difference with significantly higher accumulative low‐beta powers in healthy group (all *p* < 0.001) than MCS and UWS/VS with frontal (healthy vs. MCS: 172.77 ± 34.00 vs. 117.13 ± 58.16, *p* < 0.001; and vs. UWS/VS: 102.63 ± 37.57, *p* < 0.001), sensorimotor (healthy vs. MCS: 144.44 ± 23.89 vs. 107.27 ± 47.91, *p* = 0.001; and vs. UWS/VS: 94.36 ± 44.91, *p* < 0.001) and parietal TMS (healthy vs. MCS: 149.75 ± 24.99 vs. 94.36 ± 47.91, *p* = 0.002; and vs. UWS/VS: 104.96 ± 50.01, *p* < 0.001) (Figure 4D,E).

**FIGURE 5:**
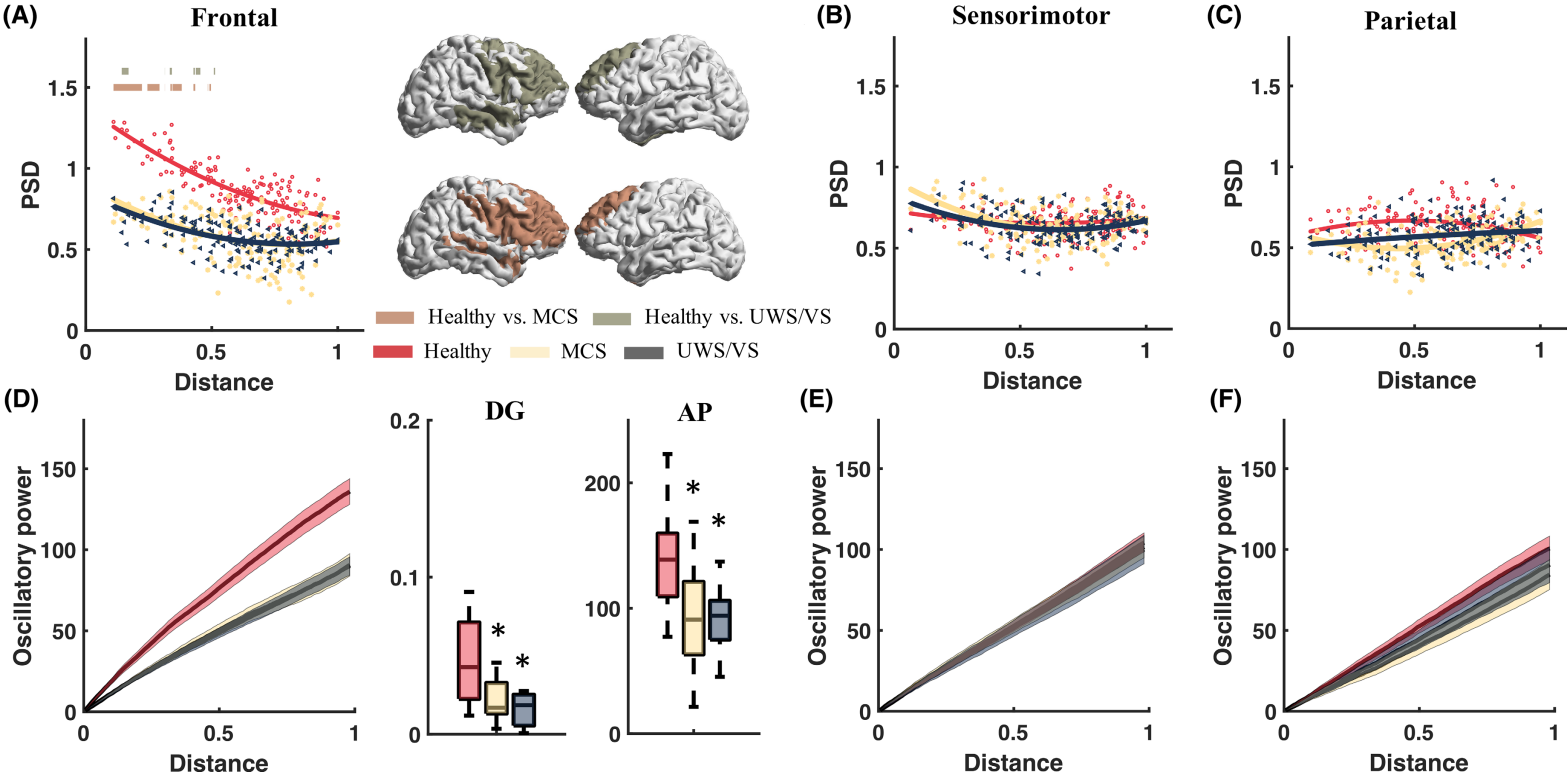
Cortical fast‐beta (21–30 Hz) reactivity to transcranial magnetic stimulation (TMS) in brain regions of healthy, minimally conscious state (MCS) and unresponsive wakefulness syndrome/vegetative state (UWS/VS). Scatter plots show fast‐beta power spectral density (PSD) of brain regions with normalized distance to stimulus targets with frontal (A), sensorimotor (B), and parietal (C) TMS of healthy (red), MCS (yellow), and UWS/VS (gray). Curves depict logistic regression between theta PSD and normalized distance. The heavy lines upper curves and spatial distribution maps at right side show statistical significance (independent two‐tails *t*‐tests with FDR correction, *p* < 0.05) in group contrasts. Accumulating fast‐beta reactivity power (mean ± SEM) along with normalized distance of brain regions to stimulus targets, with frontal (D), sensorimotor (E) and parietal (F) TMS. Boxplots show the power decay gradients (DG) and accumulative power (AP), which was significantly different between healthy subjects (red), patients with MCS (yellow), and UWS/VS (gray). * indicates significant difference (independent two‐tails *t*‐tests with FDR correction, *p* < 0.05) between healthy versus MCS or healthy versus UWS/VS.

The healthy group showed significantly (two‐tailed independent *t*‐tests, with *p* < 0.05 after FDR correction) higher power of fast‐beta reactivity in local lobes with frontal TMS, in comparison to MCS and UWS/VS groups (Figure 5A,B). Two‐way rmANOVA revealed significance of group main effects (*F*_(2, 210)_ = 8.695, *p* < 0.001) in decay gradients and significant interaction with TMS targets (*F*_(4, 210)_ = 22.816, *p* < 0.001), and significance of group main effects (*F*_(2, 210)_ = 7.216, *p* = 0.001) in accumulative fast‐beta power and interaction (*F*_(4, 210)_ = 2.278, *p* = 0.030) with TMS targets. Using TMS at frontal, healthy group had significantly higher decay gradients (healthy vs. MCS: 0.047 ± 0.027 vs. 0.022 ± 0.013; and vs. UWS/VS: 0.016 ± 0.010) and accumulative fast‐beta power (healthy vs. MCS: 135.98 ± 39.56 vs. 90.64 ± 36.53; and vs. UWS/VS: 89.91 ± 25.34) than MCS and UWS/VS groups (all *p* < 0.001). No significant difference in decay gradients and accumulative fast‐beta power between group contrasts with sensorimotor and parietal TMS.

**FIGURE 6:**
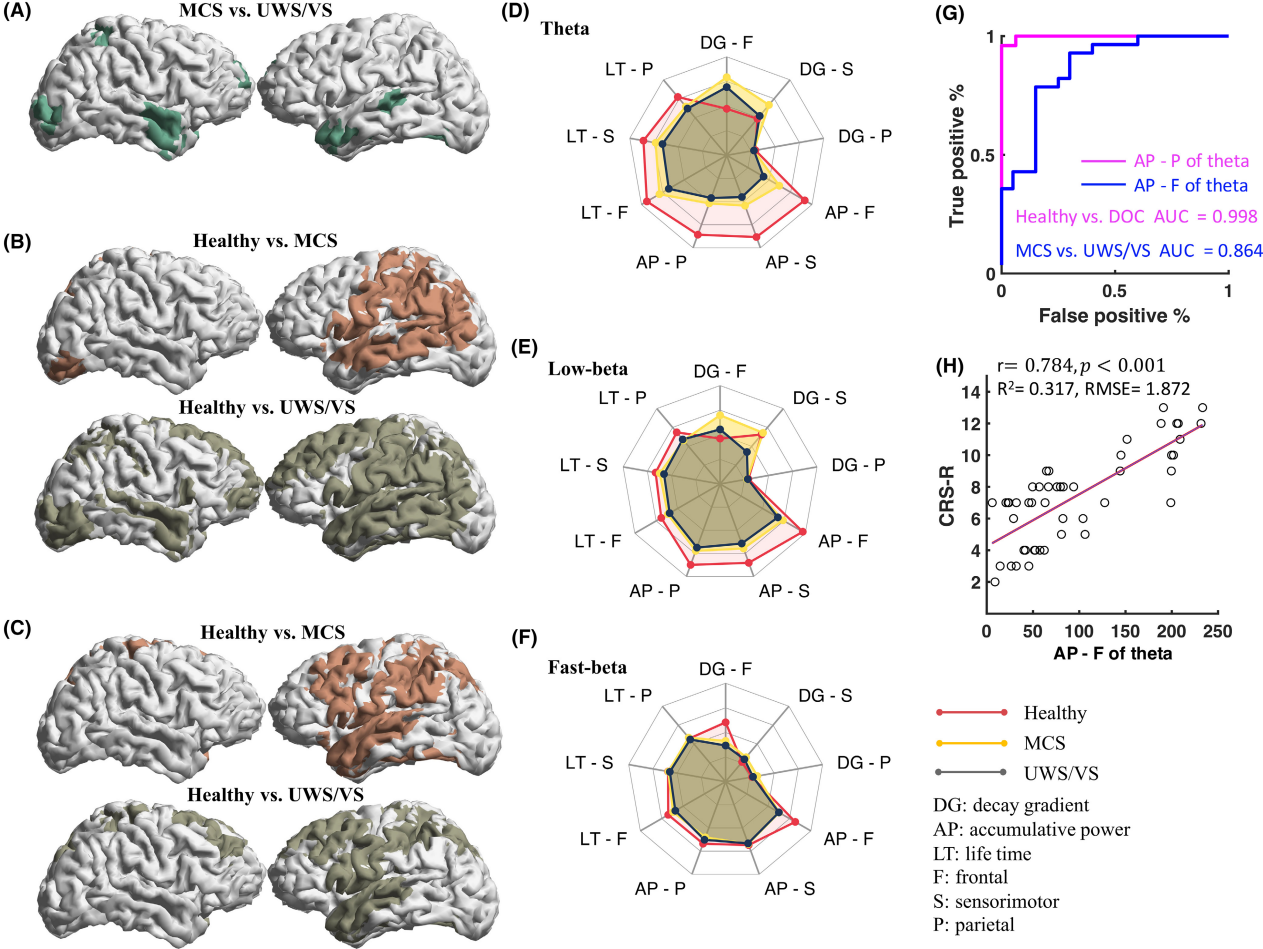
Oscillatory reactivity characteristics in healthy, minimally conscious state (MCS) and unresponsive wakefulness syndrome/vegetative state (UWS/VS). Spatial spread plots show the brain regions, which had significantly different theta reactivity between MCS versus UWS/VS, with frontal and sensorimotor transcranial magnetic stimulation (TMS) (A). Brain regions with significantly different theta (B) and low‐beta (C) reactivity between healthy controls versus MCS and UWS/VS, with frontal, sensorimotor and parietal TMS. Radar plots show group average of theta (D), low‐beta (E), and fast‐beta (F) reactivity characteristics in healthy (red), MCS (yellow), and UWS/VS (gray) groups. The characteristics include accumulative power (AP), decay gradient (DG), and life time (LT) with TMS at three targets. Values of the characteristics were normalized with maximum values among all the participants. (G) Receiver operating characteristic curves of the best properties (highest area under curves, AUC) in classifying healthy vs. disorders of consciousness (DOC) (accumulative theta reactivity power with parietal TMS, pink) and between MCS versus UWS/VS (accumulative theta reactivity power with frontal TMS, blue). (H) Generalized linear regression using the CRS‐R values as responder and theta reactivity powers (frontal TMS) as predictor. Each circle indicates one patient. Red line shows the predicted CRS‐R values using theta reactivity powers as predictor. Pearson correlation coefficients (*r* and *p*) were indicated. *R* ^2^, coefficient of determination; RMSE, root mean squared error.

Theta reactivity power in the contralateral posterior brain, including the postcentral gyrus, parietal lobules and temporal regions, was significantly different between healthy and MCS groups, across all three TMS targets. Additionally, contralateral ROIs, covering most lobes of the anterior–posterior brain, showed a significant difference in theta reactivity power between healthy and UWS/VS groups, across all three TMS targets (Figure 6B). For the DOC group contrast, ROIs covering parts of the ipsilateral middle frontal gyrus, bilateral middle temporal lobes, ipsilateral superior parietal lobes and occipital lobes, showed a significant difference in theta reactivity, across frontal and sensorimotor TMS (Figure 6A). Low‐beta reactivity power at contralateral ROIs, mainly including parts of the middle frontal gyrus, precentral and postcentral gyrus and parts of inferior parietal–temporal lobes, was significantly different between healthy vs. MCS and UWS/VS group, across TMS of all three targets (Figure 6C).

TMS‐induced oscillatory reactivity properties, particularly of life time and accumulative power at theta and low‐beta bands, showed distinct differences between healthy and DOC patients (Figure 6D,E). Fast‐beta reactivity properties, as distinguished by frontal TMS, enabled the distinction of healthy and DOC (Figure 6F). The classification between healthy and DOC patients was best achieved with the accumulative theta power of parietal TMS (AUC = 0.998), whereas the accumulative theta power of frontal TMS yielded the highest classification (AUC = 0.864) between MCS and UWS/VS patients (Figure 6G).

A stepwise logistic regression analysis revealed that the strongest predictor (*F*‐Stat = 73.20, *p* < 0.001, *R* ^2^ = 0.317, RMSE = 1.872) of CRS‐R scores was the accumulative theta power of frontal TMS (Figure 6H). Its predictive effect was confirmed by Pearson correlation (*r* = 0.784, *p* < 0.001). Wald statistic confirmed that accumulative theta power of sensorimotor TMS (Wald statistic = 17.38, *p* < 0.001), theta life time of frontal TMS (Wald statistic = 10.99, *p* = 0.002), low‐beta decay gradients of frontal (Wald statistic = 7.23, *p* = 0.010) and sensorimotor TMS (Wald statistic = 6.45, *p* = 0.015) potentially contributed to the predictive model for CRS‐R.

## 4 DISCUSSION

TMS‐EEG has been used to detect and track neural correlates of consciousness^9^ and has been used to observe a simple local response waveform in VS/UWS patients^25^ that is qualitatively similar to the one observed in unconscious healthy individuals during slow‐wave sleep^33^ or anesthesia.^34^ In MCS patients, TEP activations spread far from the stimulation site.^25^ However, the evoked/induced oscillatory reactivity, which includes both phase‐locked and non‐phase‐locked reactivity, in response to the TMS onset has not been studied in DOC. Our study compared the “total oscillatory responses” to TMS between DOC and healthy controls, and found a temporal–spatial suppression of the oscillatory reactivity in DOC patients, which may indicate a breakdown of oscillatory effective networks. Specifically, the TMS induced oscillations in DOC patients did not propagate as far away as in healthy controls, in both temporal (time to TMS pulses) and spatial (distance to TMS target sites) dimensions. Additionally, the cortical theta reactivity to TMS was found to be closely related to the residual consciousness levels of DOC patients.

Oscillatory characteristics induced by TMS in various regions of the brain have been well‐defined in recent years.^17^, ^35^ The findings of this study suggest that cortical oscillatory reactivity to TMS in healthy controls is generally dominated by three frequency bands with peaks at theta, slow‐beta and fast‐beta. Beta reactivity is a common oscillatory reactivity to TMS and has been observed in the anterior–posterior cortex. The beta reactivity observed in our study was consistent with the regional‐specific natural or resonant frequency, which reflects in a brief period of synchronization of neuronal firing at a specific frequency following the TMS pulse. Specifically, TMS has been shown to induce fast beta and gamma band oscillations in the frontal regions,^17^ beta and gamma band oscillations in the premotor cortex,^19^ and beta band oscillations in the parietal regions.^17^ In contrast, in DOC patients, although no distinct beta peaks were observed, a broad band beta (13–30 Hz) oscillation still dominated the spectrum of oscillatory reactivity (Figure 1). This suggests that the broadband beta reactivity may be an elementary cortical response in perturbation of TMS, not necessarily mediated by higher‐level brain consciousness. However, the fast‐beta response to TMS of the frontal region was significantly different (mainly located in frontal regions) between healthy controls and DOC patients, indicating a close relationship between frontal‐specific resonant beta (natural frequency) and consciousness. This is thought to reflect the local intrinsic dynamics of the corresponding corticothalamic circuits,^17^ and suggests that an abnormal frontal resonant frequency may be indicative of an injured fronto‐thalamic circuit in DOC, highlighting the important role of the fronto‐thalamic circuit in supporting human consciousness, as proposed by mesocircuit model.^2^

It has been observed that, in addition to the beta oscillation, theta oscillation is also a primary oscillatory reactivity to TMS at the target sites of healthy controls, which has not been reported in previous studies. This could be due to the methodological calculation of the natural frequency, which is usually isolated by subtracting the phase‐locked TMS evoked responses, but overlooking the phase‐locked EEG response components to the TMS pulse. However, we must consider that even a single TMS pulse can generate a complex cascade of events characterized by a certain level of variability, in terms of phase and latency. We suggest that the “total oscillatory responses” procedure, which captures both the non‐stationary and event‐locked reactivity, has a higher tolerance and can thus describe the possible transient modulations of oscillatory activity caused by TMS.^36^

The power of theta reactivity was found to be higher in MCS patients when compared to healthy controls, with a distinctly lower peak in the former. However, VS/UWS patients showed no such peak, suggesting a correlation between consciousness levels and theta reactivity. It has been hypothesized that TMS oscillatory activity is generated by the same neurophysiological process as spontaneous oscillations.^18^ This is supported by the fact that VS/UWS patients had lower spontaneous theta power than MCS patients.^9^ Additionally, the power of theta reactivity in VS/UWS patients was lower and decayed more quickly than in MCS patients in both temporal and spatial directions (Figures 2 and 3). It is thought that both spontaneous and event‐related theta activity are broadly distributed across the brain^37^ and associated with active operations of the brain cortex, particularly during high‐level cognitive processes (i.e., cognitive top‐down control)^38^, ^39^, ^40^ and internal/external attention.^41^ Studies showed that TMS‐induced theta oscillations were linked to a bottom‐up network of working memory.^42^, ^43^ Our research revealed that the spatial locations of significant differences (MCS vs. VS/UWS) in theta reactivity (Figure 6B) included middle frontal, parietal (superior parietal lobule and inferior parietal lobule), temporal and occipital regions. These regions are important hubs that make up the top‐down and bottom‐up networks, and are essential for cognitive processing and control.^38^, ^44^ The frontal and parietal cortex are necessary for sustaining and mediating attention.^45^, ^46^, ^47^ This suggests that the breakdown propagation of theta reactivity may be related to the lack of cognitive processes and attention in DOC patients. The positive correlation between the power of frontal theta reactivity and CRS‐R (Figure 6H) further supports this idea, as CRS‐R includes content related to attention, cognition and awareness.^3^

EEG oscillations have an important role in deciphering brain activity from distant areas. Generally, slow‐frequency EEG oscillations demonstrate the collective activity of large neuronal networks in the brain while high‐frequency oscillations primarily depict the activity of local neuronal populations.^48^ Thus, we hypothesize that the significant difference (MCS vs. VS/UWS) of slow (i.e., theta) oscillations, but not fast (i.e., beta) oscillations, largely reflects a breakdown of large‐scale disconnection in unconsciousness state. Furthermore, beta reactivity in remote (away from TMS sites) regions, but not local (near TMS sites) regions significantly distinguished healthy controls from DOC patients (Figure 6A). This confirms a large‐scale disruption of oscillatory networks in DOC, which is in line with previous findings from spontaneous activities.^9^, ^49^ Therefore, the frequency‐specific temporal–spatial breakdown suggests a disruption of large‐scale oscillatory effective networks with consciousness impairment. This cortical‐stimulus–response evidence highlights the association between oscillatory effective networks and human consciousness, which is consistent with the central tenets of Integrated information theory.^50^

TMS has been found to evoke cortical activation not only at the stimulated site^51^ but also in distant cortical regions.^33^, ^52^ TEPs analysis has revealed a disruption in effective connectivity in DOC patients.^25^ Our study, using TMS‐EEG to measure oscillatory responses, revealed a wide spread breakdown of oscillatory networks in DOC cortex. This oscillatory reactivity gauges the ability of groups of neurons to interact effectively to form an integrated whole for information processing.^24^ It is proposed that cortical circuits become inactive in unconsciousness, resulting in temporal and spatial suppression of cortical reactivity, disrupting local causal interactions, and preventing the build‐up of oscillatory connections in DOC cortex. Theoretical,^53^, ^54^ experimental,^55^, ^56^ and clinical evidence^57^, ^58^, ^59^ suggests that consciousness is reliant on the capacity of different regions of the brain to interact through cortico‐cortical and cortico‐thalamo‐cortical connections. Taking account of the roles of cortico‐thalamic circuits in generating oscillatory reactivity to TMS,^17^, ^60^ the results of this study suggest that the availability of effective oscillatory interactions between cortico‐thalamic modules may be an important factor in determining the presence or absence of consciousness.

We conducted a study to assess the relationship between cortical oscillatory reactivity and consciousness states in a sample of patients with a disorder of consciousness. However, due to the small sample size, it was not possible to rule out the influence of factors such as demographic or etiologic characteristics. Additionally, those without detectable MEPs were excluded, which may have skewed the results of the TMS‐EEG. Furthermore, the TMS‐EEG was conducted using neuro‐navigation with structure images rather than overlapping with brain lesions, thus not taking into account the effects of perilesional off‐periods^60^ or inexcitable cortex.^61^

Our findings demonstrate that TMS‐induced oscillatory reactivity was suppressed in temporal–spatial propagation, implying breakdown of oscillatory effective networks in DOC patients. This provides a new insight into the part of brain oscillation networks in human consciousness, and further the use of TMS‐EEG in DOC evaluation as well as our comprehension of neurophysiological foundations of human consciousness.

## CONFLICT OF INTEREST STATEMENT

The authors declare that they have no conflicts of interest.

## Supporting information


Table S1.

## Data Availability

The data and all custom written MATLAB codes that support the findings of this study are available on request from the corresponding author. The data are not publicly available due to privacy or ethical restrictions.
