# Supplementary material for: Breakdown of oscillatory effective networks in disorders of consciousness
Source: CNS Neurosci Ther. 2023 Sep 17;30(3):e14469. doi: 10.1111/cns.14469 (PMC10916448; doi:10.1111/cns.14469)
Supplement: Supplementary file 1 — Table S1. [file CNS-30-e14469-s001.docx]

Supplementary Table S1. Characteristics of the patients with disorders of consciousness.

| Number | Gender | Age (years) | Etiology | Time since injury (months) | Best CRS-R score  (total score/sub-scores) | Diagnosis |
| --- | --- | --- | --- | --- | --- | --- |
| 1 | F | 50 | Anoxia | 5 | 8/302102 | MCS |
| 2 | M | 63 | Stroke | 8.5 | 11/332102 | MCS |
| 3 | F | 40 | Trauma | 2.5 | 12/333102 | MCS |
| 4 | M | 47 | Anoxia | 3 | 13/324112 | MCS |
| 5 | F | 62 | Stroke | 10 | 8/113102 | MCS |
| 6 | M | 55 | Stroke | 3.5 | 8/113102 | MCS |
| 7 | M | 53 | Trauma | 3.5 | 10/133102 | MCS |
| 8 | M | 46 | Stroke | 5 | 8/113102 | MCS |
| 9 | M | 42 | Stroke | 5 | 8/032102 | MCS |
| 10 | M | 77 | Stroke | 2.5 | 8/032102 | MCS |
| 11 | F | 29 | Anoxia | 3 | 9/132102 | MCS |
| 12 | F | 31 | Trauma | 5.5 | 12/135102 | MCS |
| 13 | M | 54 | Stroke | 4 | 13/332212 | MCS |
| 14 | M | 36 | Trauma | 3 | 8/032102 | MCS |
| 15 | F | 70 | Stroke | 3.5 | 7/113101 | MCS |
| 16 | M | 30 | Trauma | 2 | 7/122101 | MCS |
| 17 | F | 44 | Trauma | 3 | 7/103102 | MCS |
| 18 | F | 56 | Trauma | 1 | 7/013111 | MCS |
| 19 | M | 64 | Stroke | 4 | 9/132102 | MCS |
| 20 | M | 65 | Stroke | 4 | 9/312102 | MCS |
| 21 | M | 25 | Trauma | 3 | 10/232102 | MCS |
| 22 | M | 59 | Stroke | 3 | 7/122101 | MCS |
| 23 | F | 45 | Trauma | 5 | 11/233102 | MCS |
| 24 | F | 30 | Anoxia | 11 | 9/213102 | MCS |
| 25 | M | 82 | Stroke | 4 | 7/032002 | MCS |
| 26 | F | 37 | Anoxia | 2.5 | 10/132103 | MCS |
| 27 | F | 70 | Stroke | 2.5 | 12/333102 | MCS |
| 28 | M | 30 | Trauma | 6 | 12/333102 | MCS |
| 29 | M | 38 | Anoxia | 6 | 3/001002 | UWS/VS |
| 30 | F | 74 | Stroke | 5 | 6/102102 | UWS/VS |
| 31 | F | 65 | Anoxia | 8 | 5/102002 | UWS/VS |
| 32 | M | 70 | Stroke | 6.5 | 3/001002 | UWS/VS |
| 33 | F | 62 | Stroke | 4.5 | 4/002101 | UWS/VS |
| 34 | M | 57 | Anoxia | 4 | 4/011002 | UWS/VS |
| 35 | M | 47 | Anoxia | 4.5 | 3/001002 | UWS/VS |
| 36 | M | 27 | Trauma | 7 | 3/001002 | UWS/VS |
| 37 | M | 64 | Anoxia | 6 | 5/112001 | UWS/VS |
| 38 | M | 64 | Stroke | 13 | 2/002000 | UWS/VS |
| 39 | F | 37 | Anoxia | 3 | 4/001102 | UWS/VS |
| 40 | M | 24 | Anoxia | 1.5 | 4/102100 | UWS/VS |
| 41 | F | 53 | Trauma | 8 | 6/102102 | UWS/VS |
| 42 | M | 41 | Stroke | 6 | 4/102001 | UWS/VS |
| 43 | F | 60 | Stroke | 3 | 7/112102 | UWS/VS |
| 44 | F | 34 | Trauma | 8 | 6/102102 | UWS/VS |
| 45 | F | 33 | Anoxia | 2.5 | 7/112102 | UWS/VS |
| 46 | F | 50 | Anoxia | 3 | 7/112102 | UWS/VS |
| 47 | M | 62 | Stroke | 3 | 4/002101 | UWS/VS |
| 48 | M | 26 | Trauma | 3 | 7/112102 | UWS/VS |

F - female; M - male; CRS-R - Coma Recovery Scale-Revised; MCS - minimally conscious state; UWS/VS - unresponsive wakefulness syndrome / vegetative state.
